# Diversity and ethics in trauma and acute care surgery teams: results from an international survey

**DOI:** 10.1186/s13017-022-00446-8

**Published:** 2022-08-10

**Authors:** Lorenzo Cobianchi, Francesca Dal Mas, Maurizio Massaro, Walter Biffl, Fausto Catena, Federico Coccolini, Beatrice Dionigi, Paolo Dionigi, Salomone Di Saverio, Paola Fugazzola, Yoram Kluger, Ari Leppäniemi, Ernest E. Moore, Massimo Sartelli, George Velmahos, Sarah Woltz, Peter Angelos, Luca Ansaloni, the Team Dynamics Study Group, Abubaker Abdelmalik, Nebyou Seyoum Abebe, Fikri M. Abu-Zidan, Yousif Abdallah Yousif Adam, Harissou Adamou, Antonino Agrusa, Emrah Akin, Henrique Alexandrino, Syed Muhammad Ali, Pedro Miguel Almeida, Francesco Amico, Michele Ammendola, Jacopo Andreuccetti, Daniel Aparicio-Sanchez, Antonella Ardito, Giulio Argenio, Ingolf Harald Askevold, Boyko Tchavdarov Atanasov, Goran Augustin, Selmy Sabry Awad, Carlo Bagnoli, Lovenish Bains, Dimitrios Balalis, Edoardo Baldini, Oussama Baraket, Mirko Barone, Jorge Arturo Barreras, Giovanni Bellanova, Helena Biancuzzi, Mark Brian Bignell, Roberto Bini, Daniele Bissacco, Paoll Boati, Andrea Bottari, Konstantinos Bouliaris, Antonio Brillantino, Luis Antonio Buonomo, Salvatore Buscemi, Valentin Calu, Riccardo Campo Dall’Orto, Stefano Campostrini, Joao Miguel Carvas, Gianmaria Casoni Pattacini, Valerio Celentano, Marco Ceresoli, Mircea Chirica, Pasquale Cianci, Nicola Cillara, Stefania Cimbanassi, Stefano Piero Bernardo Cioffi, Elif Colak, Luigi Conti, Silvia Dantas Costa, Fabrizio D’Acapito, Dimitrios Damaskos, Koray Das, Richard Justin Davies, Andrew Charles De Beaux, Belinda De Simone, Zaza Demetrashvili, Andreas Kyriacou Demetriades, Stefano Denicolai, Giuseppe Di Buono, Isidoro Di Carlo, Bogdan Diaconescu, Rigers Dibra, Sandra Dios-Barbeito, Agron Dogjani, Maurizio Domanin, Mario D’Oria, Virginia Duran Munoz-Cruzado, Barbora East, Gerald Takem Ekwen, Adel Hamed Elbaih, Juan Pablo Escalera-Antezana, Giuseppe Esposito, Roser Farre, Antonjacopo Ferrario di Tor Vajana, Vinicius Cordeiro Fonseca, Francesco Forfori, Laura Fortuna, Evangelos Fradelos, Gustavo P. Fraga, Pietro Fransvea, Mahir Gachabayov, Alain A. Garcia Vazquez, Wagih Mommtaz Ghannam, Rossella Gioco, Giorgio Giraudo, Mario Giuffrida, Michela Giulii Capponi, Carlos Augusto Gomes, Ricardo Alessandro Teixeira Gonsaga, Emre Gonullu, Jacques Goosen, Tatjana Goranovic, Ewen Alexander Griffiths, Muad Gamil Haidar, Hytham K. S. Hamid, Timothy Craig Harddastle, Andrew J. Healey, Matthias Hecker, Edgar Fernando Hernandez Garcia, Eduardo Cancio Huaman, Martin Hutan, Orestis Ioannidis, Arda Isik, Azzain Mahadi Hamid Ismail, Nizar Ismail, Ji Young Jang, Haytham M. A. Kaafarani, Sujala Niatarika Rajsain Kalipershad, Lewis J. Kaplan, Yasin Kara, Evika Karamagioli, Aleksandar Karamarkovia, Alfie J. Kavalakat, Aristotelis Kechagias, Jakub Kenig, Jim S. Khan, Vladimir Khokha, Roberto Klappenbach, Yoshiro Kobe, Victor Kong, Dimitrios Korkolis, Hayato Kurihara, Akira Kuriyama, Aitor Landaluce-Olavarria, Leo Licari, Andrey Litvin, Tyler J. Loftus, Varut Lohsiriwat, Claudia Cristina Lopes Moreira, Eftychios Lostoridis, Agustín Tovar Luna, Davide Luppi, Gustavo Miguel Machain, Marc Maegele, Daniele Maggiore, Ronald V. Maier, Mallikarjuna Manangi, Andrea Manetti, Baris Mantoglu, Federico Mariani, Athanasios Marinis, Evandro Antonio Sbalcheiro Mariot, Giuseppe Roberto Marseglia, Jacopo Martellucci, Gennaro Martines, Aleix Martinez Perez, Pietro Mascagni, Damien Massalou, Renato Bessa Melo, Luca Miceli, Andrea Mingoli, Tushar S. Mishra, Ali Yasen Y. Mohamedahmed, Rajashekar Mohan, Dieter Morales-Garcia, Sami Mohamed Siddig Mustafa, Mukhammad David Naimzada, Ionut Negoi, Christine Nguyen, Melkamu Kibret Nidaw, Giuseppe Nigri, Habeeb Damilola Ogundipe, Cristina Oliveri, Stefano Olmi, Leonardo Pagani, Giuseppe Palomba, Desire Pantalone, Arpad Panyko, Ciro Paolillo, Davide Papis, Nikolaos Pararas, Francesco Pata, Simon Paterson-Brown, Giovanna Pavone, Francesca Pecchini, Gianluca Pellino, Maria Pelloni, Andrea Peloso, Eduardo Perea del Pozo, Rita Goncalves Pereira, Bruno Monteiro Pereira, Aintzane Lizarazu Perez, Gennaro Perrone, Antonio Pesce, Giovanni Petracca, Micaela Piccoli, Daniele Piccolo, Edoardo Picetti, Emmanouil Pikoulis, Tadeja Pintar, Giovanni Pirozzolo, Mauro Podda, Pietro Previtali, Francesca Privitera, Clelia Punzo, Martha Alexa Quiodettis, Niels Qvist, Razrim Rahim, Alexander Reinisch-Liese, Maria Rita Rodriguez-Luna, Daniel Roizblatt, Francesco Pietro Maria Roscio, Stefano Rossi, Federico Ruta, Boris Evgeniev Sakakushev, Juan Carlos Salamea, Ibrahima Sall, Fabrizio Sammartano, Alejandro Sanchez Arteaga, Sergio Sanchez-Cordero, Diego Sasia, Robert G. Sawyer, Charalampos Seretis, Mario Serradilla-Martin, Vishal G. Shelat, Sergei Shlyapnikov, Romeo Lages Simoes, Boonying Siribumrungwong, Mihail Slavchev, Leonardo Solaini, Gabriele Soldini, Kjetil Soreide, Florian Spada, Philip Stahel, Larysa Sydorchuk, Ruslan Sydorchuk, Ali Muhammad Syed, Luis Tallon-Aguilar, Jih Huei Tan, Edward Tan, Antonio Tarasconi, Dario Tartaglia, Nicola Tartaglia, John Taylor, Giovanni Domenico Tebala, Ricardo Alessandro Teixeira Gonsaga, Michel Teuben, Matti Tolonen, Giovanni Tomasicchio, Tania Triantafyllou, Giuseppe Trigiante, Victor Turrado-Rodriguez, Roberta Tutino, Matteo Uccelli, Bakarne Ugarte-Sierra, Mika Ukkonen, Panteleimon G. Vassiliu, Juan Manuel Verde, Massimiliano Veroux, Ramon Vilallonga, Diego Visconti, Maciej Waledziak, Tongporn Wannatoop, Lukas Werner Widmer, Michael Samuel James Wilson, Ting Hway Wong, Sofia Xenaki, Byungchul Yu, Steven Yule, Andee Dzulkarnaen Zakaria, Diego A. Zambrano, Monica Zese

**Affiliations:** 1 Department of Clinical, Diagnostic and Pediatric Sciences, University of Pavia, Via Alessandro Brambilla, 74, 27100 Pavia, PV Italy; 2 Department of General Surgery, IRCCS Policlinico San Matteo Foundation, Pavia, Italy; 3 Department of Management, Ca’ Foscari University of Venice, Venice, Italy; 4 Division of Trauma and Acute Care Surgery, Scripps Memorial Hospital La Jolla, La Jolla, CA USA; 5 Bufalini Hospital, AUSL Romagna, Cesena, Italy; 6 General, Emergency and Trauma Surgery Departmrnt, Pisa University Hospital Pisa, Pisa, Italy; 7 New York Presbyterian Columbia University Irvine Medical Center, Irvine, NY USA; 8 ASUR Marche 5, San Benedetto del Tronto General Hospital, San Benedetto del Tronto, Italy; 9 Division of General Surgery, Rambam Health Care Campus, Haifa, Israel; 10 Abdominal Center, Helsinki University Hospital and University of Helsinki, Helsinki, Finland; 11 Department of Surgery, Denver Health Medical Center, Denver, CO USA; 12 Department of Surgery, Macerata Hospital, Macerata, Italy; 13 Harvard Medical School, Boston, MA USA; 14 Division of Trauma, Emergency Surgery, and Surgical Critical Care, Massachusetts General Hospital, Boston, MA USA; 15 Department of Surgery, Spaarne Gasthuis, Hoofddorp, The Netherlands; 16 Department of Surgery and MacLean Center for Clinical Medical Ethics, The University of Chicago, Chicago, IL USA

**Keywords:** Trauma surgery, Ethics, Acute care surgery, Diversity, Team dynamics

## Abstract

**Background:**

Investigating the context of trauma and acute care surgery, the article aims at understanding the factors that can enhance some ethical aspects, namely the importance of patient consent, the perceptiveness of the ethical role of the trauma leader, and the perceived importance of ethics as an educational subject.

**Methods:**

The article employs an international questionnaire promoted by the World Society of Emergency Surgery.

**Results:**

Through the analysis of 402 fully filled questionnaires by surgeons from 72 different countries, the three main ethical topics are investigated through the lens of gender, membership of an academic or non-academic institution, an official trauma team, and a diverse group. In general terms, results highlight greater attention paid by surgeons belonging to academic institutions, official trauma teams, and diverse groups.

**Conclusions:**

Our results underline that some organizational factors (e.g., the fact that the team belongs to a university context or is more diverse) might lead to the development of a higher sensibility on ethical matters. Embracing cultural diversity forces trauma teams to deal with different mindsets. Organizations should, therefore, consider those elements in defining their organizational procedures.

**Level of evidence:**

Trauma and acute care teams work under tremendous pressure and complex circumstances, with their members needing to make ethical decisions quickly. The international survey allowed to shed light on how team assembly decisions might represent an opportunity to coordinate team member actions and increase performance.

## Background

Trauma is the leading cause of death among people aged 1–44 years and the fourth most common cause of death in the western world [1]. When managing trauma cases, the member of an emergency and trauma surgical team must work under tremendous pressure in a stressful and complex environment while aiming to communicate effectively, and decisions must often address ethical dilemmas [2]. Indeed, trauma teams must deal with extreme stress and time constraints, not rarely with no awareness of the trauma causes nor the patients’ identity, current circumstances or conditions, or patients’ desires about the treatment options, and must cope with the risk of unanticipated incidents. Within this context, traditional organizational practices are less effective, and integrative mechanisms such as team assembly decisions might be the only opportunity to coordinate team member actions and increase performance [3].

In such scenarios, urgent decision-making in trauma surgery raises a number of ethical concerns. When faced with ethical dilemmas, clinicians must rapidly consider the potential outcomes of their choices, as well as the limited information they have about their patients [4]. Ethics also involves communication and sharing of eventual options and decisions to patients and their loved ones with consistency and compassion. The emergency situation rarely allows time to investigate recommendations from the relevant literature or guidelines or ask other colleagues for second opinions. As a result, there is a high risk of making mistakes or facing ethical dilemmas when managing injured patients, and this seems to be particularly true during the early assessment and resuscitation processes when lifesaving procedures are made [5]. Similarly, the time or opportunity to inform the patient might be limited or completely lacking. In this perspective, the topic of consent is critical. Because of the sudden and unexpected nature of trauma or the emergency, healthcare providers must be trained to think and respond fast in the patient's best medical interest. Trauma patients frequently have transitory impairments in their ability to make autonomous and informed decisions. This often results in *presumed consent* for medically necessary treatment [4].

Emergency surgery teams are made up of a multidisciplinary group of people from different specialities such as anesthesia, emergency medicine, surgery, nursing, and supporting staff, all of whom provide simultaneous inputs into the trauma assessment and treatment, with a team leader who will be coordinating their activities [1]. Therefore, in trauma and emergency situations, team dynamics are critical [6].

A crucial aspect refers to the role of the trauma leader who, as an ethical leader and guided by strong ethical principles, “can experience conflicting obligations to stakeholders during these emotional and complex debates and must lead with strength, compassion, fairness, and justice while eliminating implicit and explicit biases” [7]. Indeed, while decision-making is often seen through the lens of an individual's internal cognitive process, the ability to put certain decisions into action necessitates the ability to seamlessly organize all team members to achieve the desired objective [5]. Trauma leaders have the challenging role of managing their team through potentially challenging ethical situations.

Interestingly, current teaching and assessment approaches for these advanced cognitive skills are subjective, lack standardization, and are vulnerable to errors [5, 6]. Therefore, knowledge translation and knowledge transfer mechanisms are cornerstones to allow team members to bridge their differences and communicate effectively, boosting the potential of multidisciplinary teams [8]. Fostering a continuous learning requirement on these matters is essential to enable trauma teams to overcome ethical dilemmas in an efficient and effective way [9].

Diversity in surgery is a rising topic within the surgical community. It has been defined as “a broad representation of viewpoints, socioeconomic backgrounds, gender, sexual orientation, disability status, race, and ethnicity” [10]. Emergency surgery teams are diverse by definition, as they include professionals of different specialities and variable backgrounds [1], who do need interpersonal skills [5, 6] to ensure effective care to trauma patients. The literature highlights how global performance has been shown to be better when the environment of medical care is diverse, and patient satisfaction is also improved in such contexts [11, 12]. Still, recent studies have also underlined how diversity needs dedicated organizational and management policies to reach its full potential [8, 11, 13]. Interestingly, to the best of our knowledge, little has been said about the possible influence between diversity and ethics in emergency and trauma teams.

To fill this gap, this study aims at investigating the different perceptions of trauma surgeons on three ethical-related matters. First, the study investigates the feelings of surgeons regarding the importance of patient consent. Second, the research focuses on their perceptiveness of the ethical role of the trauma leader. Third, the study deepens the perceived importance of ethics as an educational subject. This study builds on these foundations by undertaking an international survey under the auspices of the World Society of Emergency Surgery (WSES) to investigate the factors that can enhance these three ethical aspects in trauma and emergency surgery. The dataset is analyzed focusing on the role of gender, kind of institution (academic or not), and membership to an officially set trauma team and to a diverse group.

## Methods

### Survey settings and data collection

An online population-based survey was used to gather demographic, experience, and practice-based information about the trauma surgeons participating in the study and their team dynamics.

The survey was performed using Google Forms in English and reported according to the CHERRIES (Checklist for Reporting Results of Internet E-Surveys) methodology [14]. Considering the characteristics and aim of the study and the participants, the approval of the Institutional Review Board (IRB) was not needed.

The electronic questionnaire was developed following a research protocol that was circulated among the steering committee members and tested by a sample of surgeons. The majority of the questions were based on previous research in trauma and emergency surgery [9, 15, 16], information management and organization science [17–19], and medical ethics [7–9, 20].

The WSES sent out an e-mail invitation to all its 917 members in January 2021. The call for participation in the survey was also posted on the society's Web site and Twitter account. The survey's topic and objectives were detailed in the invitation e-mail, along with the survey's estimated length (less than 15 min) and the option to join the Team Dynamics Study Group to continue investigating and sharing the findings. Both of the answers, as well as the identity of the investigators, were kept confidential. Three reminders were sent out during the opening time of the investigation, which lasted one month.

### Survey design and questions

The first questions focused on identifying the sample, such as gender, years of trauma surgery experience, type of institution (academic vs. non-academic), country, the role held, eventual participation within a trauma team (meaning, a designated team within the hospital or institution which can be freed up from the routinary tasks to receive trauma patients when needed [21]), and the involvement of diverse team members. More in detail, surgeons were not given any definition of “diversity” or “diverse team.” They were just asked to declare if they considered their team as diverse. The majority of the questions asked came from Woltz et al. [15] and Reichert et al. [16].

Ethics was investigated through 12 sentences to be rated according to a five-point Likert scale, where 1 = strongly disagree and 5 = strongly agree. The items were adapted from Angelos et al. [7], Angelos [20], Suah and Angelos [4] and Scarlet [9], aiming at measuring the topics of surgical consent (items 1–2), the formal attention to ethics in trauma surgery training (items 3–7), and the role of trauma leaders as ethical leaders (items 8–12). Appendix 1 reports the items for each topic. The complete survey is included in Appendix 2.

### Data analysis

The final dataset was downloaded into an excel spreadsheet file shortly after the investigation was completed, obtaining a total of 402 fully completed questionnaires out of 917 members enrolled in the WSES (45.84% as a global response rate). Summary statistics were used to evaluate quantitative data. Results were analyzed using the software R [22].

A first analysis was developed to describe the sample distribution in terms of gender, type of institutions, respondents belonging to a trauma team, and a diverse team. Additionally, descriptive statistics were developed on the 12 ethical questions. Finally, questions were grouped into three main latent variables. Kruskal–Wallis analysis was used to check for differences in respondents by gender, kind of institution, membership to a trauma team, and a team qualified by the surgeons as diverse.^1^

## Results

### Sample distribution

For the analysis, 402 valid questionnaires were obtained. Regarding the geographical location, although the majority of the survey's participants work in Europe, the respondents are well spread around the world, representing 72 countries on all the continents. The sample shows a predominance of male surgeons (84%) compared to women (15%), with only three respondents who preferred not to answer the question. Academic institutions are the most frequent (72%), as well as the participants belonging to trauma teams (79.6%). Interestingly, 62% of the respondents declared to be working within a diverse team. Table 1 reports the descriptive statistics of the participants of the investigation.

**Table 1:** Descriptive statistics about surgeons and institutions participating in the study

	Number	Percent (%)
Participants	402	100.00
Gender		
Male	338	84.08
Female	61	15.17
Prefer not to answer	3	0.75
Kind of institution		
Academic	292	72.64
Non-academic	110	27.36
Part of a trauma team		
Yes	320	79.60
No	82	20.40
Part of a diverse team		
Yes	250	62.19
No	152	37.81

### Descriptive statistics

Focusing on the investigated 12 items, findings show an average result ranging from a minimum of 2.83 to a maximum of 4.42. Applying a Cronbach’s alpha analysis, answers' results demonstrate that questions show an internal consistency, namely how closely related a set of items are as a group, since all the replies show a Cronbach value equal or higher than 0.7 (Table 2).

**Table 2:** Descriptive statistics about the answers received

Statistic	*N*	Mean	St. Dev	Min	Pctl(25)	Median	Pctl(75)	Max	Cronbach’s alpha
*Importance of consent*									0.70
Quest1	402	2.83	1.21	1	2	3	4	5	
Quest2	402	3.48	1.16	1	3	4	4	5	
*Trauma leader as ethical leader*									0.81
Quest3	402	3.96	0.96	1	3	4	5	5	
Quest4	402	4.42	0.79	1	4	5	5	5	
Quest5	402	4.35	0.80	1	4	5	5	5	
Quest6	402	4.07	0.89	1	4	4	5	5	
Quest7	402	3.58	1.08	1	3	4	4	5	
*Importance of ethical training*									0.76
Quest8	402	4.12	0.99	1	4	4	5	5	
Quest9	402	3.16	1.20	1	2	3	4	5	
Quest10	402	3.75	1.14	1	3	4	5	5	
Quest11	402	4.08	1.04	1	4	4	5	5	
Quest12	402	3.25	1.22	1	2	3	4	5	

### Differences in respondents

Analyzing differences among respondents, our findings can be summarized as follows. First, the importance of consent is considered essential by all the respondents with no differences in terms of gender. Interestingly, respondents belonging to academic institutions showed greater attention on the topic with an average of 3.238 against an average of 2.827 of non-academics (*p* value 0.008). Similarly, respondents belonging to a structured trauma team are used to pay closer attention to this topic with an average of 3.212 against an average of 2.921 of participants not belonging to trauma teams (*p* value 0.037). Finally, surgeons who declared to belong to diverse teams underline greater attention on the importance of consent with an average of 3.328 against an average of 2.865 (*p* value > 0.001) (Table 3).

**Table 3:** Importance of consent

Statistic	*N*	Average (SD)	Median (*Q*1–*Q*3)	Kruskal–Wallis rank-sum test	
				Chi-square	*p* value
*Gender*				0.262	0.877
Female	61	2.770 (0.992)	3 [2–4]		
Prefer not to answer	3	3.000 (1.528)	3 [2.5–4]		
Male	338	2.834 (1.043)	3 [2.5–4]		
*Kind of institution*				6.944	0.008
Academic	292	3.238 (1.019)	3.5 [2.5–4]		
Non-academic	110	2.927 (1.051)	3 [2–3.5]		
*Part of a trauma team*				4.308	0.037
No	82	2.921 (1.118)	3 [2–3.875]		
Yes	320	3.212 (1.007)	3 [2.5–4]		
*Part of a diverse team*				18.746	> 0.001
No	152	2.865 (1.070)	3 [2–3.5]		
Yes	250	3.328 (0.976)	3.5 [2.5–4]		

Focusing on the trauma leader's role as an ethical leader, results do not show statistically relevant differences for gender and membership of academic or non-academic institutions. While female surgeons get an average of 3.279 and mean 3.288, similarly, academics show an average of 3.306 and non-academics 3.224, all with *p* values higher than the statistical significance rate of 0.005. Differently, results demonstrated statistically significant differences both between respondents belonging or not to trauma teams (with means of 3.312 and 3.171, respectively, and a *p* value of 0.032) and as part of diverse teams (with means of 3.370 and 3.141, respectively, with a *p* value of less than 0.001) (Table 4).

**Table 4:** Trauma leader as an ethical leader

Statistic	*N*	Average (SD)	Median (*Q*1–*Q*3)	Kruskal–Wallis rank-sum test	
				Chi-square	*p* value
*Gender*				2.294	0.317
Female	61	3.279 (0.595)	3.2 [3–3.8]		
Prefer not to answer	3	2.867 (0.416)	3 [2.7–3.1]		
Male	338	3.288 (0.565)	3.4 [3–3.8		
*Kind of institution*				1.961	0.161
Academic	292	3.306 (0.566)	3.4 [3–3.8]		
Non-academic	110	3.224 (0.574)	3.2 [3–3.6]		
*Part of a trauma team*				4.562	0.032
No	82	3.171 (0.571)	3.2 [3–3.6]		
Yes	320	3.312 (0.565)	3.4 [3–3.8]		
*Part of a diverse team*				7.861	> 0.001
No	152	3.141 (0.576)	3.2 [2.8–3.6]		
Yes	250	3.370 (0.547)	3.4 [3–3.8]		

Finally, focusing on the importance of ethical training, most respondents agree that ethical training is essential. Interestingly, while results do not show differences between groups in terms of gender, not surprisingly ethical training is better considered by respondents belonging to academic institutions with an average of 3.742 against an average of 3.480 of others (*p* value 0.005). Similarly, respondents being part of trauma teams declared higher importance with an average of 3.734 and *p* value of 0.001. Finally, surgeons belonging to diverse trauma teams expressed greater attention to the importance of ethical training with an average of 3.830 and a *p* value less than 0.001 (Table 5).

**Table 5:** Importance of ethical training

Statistic	*N*	Average (SD)	Median (*Q*1–*Q*3)	Kruskal–Wallis rank-sum test	
				Chi-square	*p* value
*Gender*				2.455	0.293
Female	61	3.695 (0.722)	3.8 [3.2–4.2]		
Prefer not to answer	3	3.133 (0.306)	3.2 [3–3.3]		
Male	338	3.671 (0.811)	3.8 [3.2–4.35]		
*Kind of institution*				7.644	0.005
Academic	292	3.742 (0.755)	3.8 [3.2–4.4]		
Non-academic	110	3.480 (0.869)	3.6 [3–4.2]		
*Part of a trauma team*				11.356	0.001
No	82	3.424 (0.747)	3.5 [2.85–4]		
Yes	320	3.734 (0.796)	3.8 [3.2–4.4]		
*Part of a diverse team*				22.296	> 0.001
No	152	3.408 (0.895)	3.6 [2.8–4]		
Yes	250	3.830 (0.682)	3.9 [3.4–4.4]		

## Discussion

Our results aim to understand the factors that can influence how three ethical matters are perceived in trauma surgery teams. Through the analysis of 402 fully filled questionnaires by surgeons working in 72 different countries, three main ethical topics were investigated through the lens of gender, membership of an academic or non-academic institution, of a trauma team, and a diverse group.

Regarding the importance of obtaining consent from the injured patient, results highlight greater attention paid by surgeons belonging to academic institutions, official trauma teams, and diverse groups. The topic of surgical consent has been changing its meaning over time. While once it was considered more as a legal and administrative duty before the operation, the recent literature has underlined how informed consent is an essential tool to communicate with the patient, understand his or her wishes and concerns [4, 20, 23] toward the relevance of a shared decision-making [15]. In this perspective, the more recent paradigm sees the surgeons needing to “speak less and listen more” [20] to ensure better knowledge translation with their patients [8]. This may shine a light to ethical issues in trauma and emergency surgery [4], as understanding the patient’s wishes is not always feasible.

Surgeons working in academic environments and belonging to trauma teams pay greater attention to the topic of consent. This may depend on the patient-centric organizational culture and ongoing debate concerning patients’ consent which appears to be more assertive in academic institutions and within formalized trauma teams. Diverse groups record outstanding results rather than those from non-diverse ones. While diversity in practice may be challenging due to the differences among members, it has been proven to lead to exceptional outcomes. It may be reasonable that professionals who are already focused on bridging the difference with their peers are likely to do so even with their patients.

The second topic is the role of the trauma leader as an ethical leader, able to merge strong ethical principles within his or her clinical decision-making. Those belonging to formalized trauma teams and diverse groups are keener about the importance, for a trauma leader, to include ethics in his/her role. Once again, surgeons of diverse teams gave the highest evaluation in terms of their agreement. Therefore, we can claim that those actively engaging with diverse groups of people feel that ethics is crucial in ensuring smooth team dynamics and better patient outcomes.

Ethical training is the third topic analyzed, highlighting similar results. While, generally speaking, most surgeons recognize the importance of getting proper ethical training, participants from academic institutions, formalized trauma teams, and diverse groups show more significant interest. Again, the surgical literature has underlined the need to employ training related to non-technical skills [24–27], with ethics belonging to such skills [18]. Scientific societies have taken the lead on such topics. Therefore, without surprise, those closer to this debate (namely academics and formalized trauma team members) seem to be more focused on recognizing this as a relevant issue. Non-technical skills have proven to be essential to foster knowledge translation dynamics within teams, especially when groups can be defined as diverse [8, 19]. Again, diversity seems to stimulate the need for ethical training, which is considered a central element.

Surgeons have been stereotyped as “abrasive, arrogant and difficult to work with” [28, 29], and those simplified images have influenced the way they interact with colleagues and patients. Informed consent is the ethical expression of respect for the patient’s autonomy. While previous studies showed that physicians in general no longer make paternalistic decisions for patients, our results show that less diverse trauma teams might pay less attention to patient wishes.

### Limitations and bias

This study is not without limitations. Among these, although our response rate may be considered satisfactory, our investigation may suffer from selection bias [30]. Indeed, our sample may be unrepresentative of the entire Trauma community, and this may impact the goals of our survey research. For instance, the academic settings (to which many of our participants belong) may be more sensitive toward the current debate on the topics of multidisciplinarity, diversity, and inclusion. Moreover, although we cover 72 different countries on all the continents, respondents are concentrated in some geographical areas than others [2]. Cultural and technical aspects (like the medical background of the trauma leader, e.g., as a surgeon or an anesthesiologist) and the massive presence or not of specific phenomena (like gun violence) may also affect our results.

## Conclusions

Our study has highlighted how embracing cultural diversity forces trauma teams to deal with different mindsets. While previous studies have shown that different cultures (e.g., more physician-centric vs patient-centric) can coexist within organizations [31], our results underline that some organizational factors (e.g., the fact that the team belongs to a university context or is more diverse) might lead to the development of a higher sensibility on patient consent and ethical matters. Organizations willing to promote a more patient-centric view with trauma teams focused on ethical issues should consider those elements in defining their organizational procedures. Indeed, in the context of trauma teams, where time pressure and timely action are the normal conditions rather than an exception, traditional coordination mechanisms are less effective, and teams must rely more on different strategies and tools for coordinating their actions. According to our results, integrative mechanisms such as team composition might work effectively, facilitating ethical behaviors and a more patient-oriented culture.

Qualitative studies may further deepen such dynamics, defining and sharing best practices to enable trauma teams to handle ethical issues in the best possible way.

## Data Availability

The datasets used and/or analyzed during the current study are available from the corresponding author upon reasonable request.

## Appendix 1

### Items related to ethics

#### Lack of surgical consent

1. In an emergency situation, obtaining informed consent from a traumatically injured patient is important.
2. Patients cannot be expected to make informed decisions in the midst of a trauma resuscitation and therefore the wishes of trauma patients should not be respected.

#### Trauma leaders as ethical leaders

3. Ethics influences decision-making
4. Transparency is a key value that a trauma leader should employ
5. Information management is a key value that a trauma leader should employ
6. A trauma leader is able to identify eventual ethical breaches
7. When ethical issues arise, it is clear how a trauma leader should behave

#### Formal attention to ethics in trauma surgery training

8. The formal attention to ethics should be an important component of the training and education of trauma surgeons.
9. I received adequate education in ethics during my training and education to become a trauma surgeon.
10. I would have liked to receive more formal ethics teaching during my training to become a trauma surgeon.
11. Ethics should be part of the trauma surgical curriculum
12. Philosophy may help when it comes to trauma training

## Appendix 2

### Survey*

- **0. Do you want to join the Team Dynamics study group to be included in the final scientific paper?**
  ***please select one***
  1. Yes
  2. No
- **0. If you replied yes to question #1, please type your email address**
  ***please type***
- **1. What is your gender?**
  ***please select one***
  1. Male
  2. Female
  3. Prefer not to answer
- **2. How many years of experience in Trauma Surgery do you have?**
  ***please select the number***
- **3. What kind of institution do you work for?**
  ***please select one***
  1. Academic
  2. Non-academic
- **3. What is your current position?**
  ***please select one***
  1. Resident
  2. Board-certified surgeon
  3. Senior consultant
  4. Head of the department
- **5. Are you part of a Trauma Team?**
  ***please select one***
  3. Yes
  4. No
- **6. If yes (4.), is the Trauma Team institutionalized?**
  ***please select one***
  1. Yes
  2. No
- **7. If yes (4.), what kind of Trauma Team is yours?**
  ***please select one***
  1. Mono-disciplianary
  2. Multidisciplinary
  3. Other (***please type***)
- **8. Who acts as the Trauma Leader?**
  ***please select one***
  1. A surgeon
  2. An anesthesiologist/intensivist
  3. Emergency physician
  4. An orthopedic
  5. Other (***please type***)
- **9. Did you attend any of the following courses?**
  *** Select all that apply***
  1. Fellowship
  2. Master
  3. ATLS
  4. ATOM
  5. No one
  6. Other (***please type***)
- **10. In which country do you work?**
  ***List of countries***
- **11. What does knowledge translation mean to you?**
  ***please type***
- **12. How much are the following twelve items essential for facilitating the work within Trauma Teams?**
  ***A Likert five-point scale where 1 = not relevant al all; 5 = very relevant***
  1. Colleagues trust each other
  2. Colleagues deal constructively and carefully with the information provided by others
  3. The information received is accurate all the times
  4. Colleagues have friendly relationships
  5. Colleagues have a strong sense of belonging to the team
  6. Everyone’s opinion is highly valued within the team
  7. All team members have a lot of valuable information that is interesting for the team
  8. All team members have expertise and skills that are relevant for the team
  9. Everyone’s knowledge has contributed to increasing productivity within the team
  10. Most of times, the performance of the team is as good as high level trauma centers
  11. Most of times, clinical results are in line of what can be expected from the literature concerning such cases
  12. Most of times, clinical outcomes are as initially forecast
- **13. How much are the following ten items essential for facilitating the work within Trauma Teams?**
  ***A Likert five-point scale where 1 = not relevant al all; 5 = very relevant***
  1. Ideas creation ability
  2. Coordination ability
  3. Multicultural ability
  4. Planning ability
  5. Learning ability
  6. Professionalism
  7. Leadership
  8. Information management ability
  9. Ethics
  10. Communication ability
- **14. Are non-technical skills crucial for Trauma Teams?**
  ***please select one***
  1. Yes
  2. No
- **15. Why?**
  ***please type***
- **16. Which are the tools that, in your opinion, may facilitate the work within Trauma Teams?**
  ***A Likert five-point scale where 1 = not relevant al all; 5 = very relevant***
  1. Mobile electronic Medical records and online tools
  2. Training
  3. Networking and international experiences
  4. Multidisciplinary committees and meetings
  5. Publications
  6. Clinical guidelines and cases
  7. Patients’ and stakeholders’ engagement
  8. Non-technical skills
- **17. Which of these tools do you effectively use in your daily practice within Trauma Teams?**
  *** Select all that apply***
  1. Mobile electronic Medical records and online tools
  2. Training
  3. Networking and international experiences
  4. Multidisciplinary committees and meetings
  5. Publications
  6. Clinical guidelines and cases
  7. Patients’ and stakeholders’ engagement
  8. Non-technical skills
- **18. Would you describe your Team as diverse?**
  ***please select one***
  1. Yes
  2. No
- **19. To what extent do you agree with the following statements?**
  ***A Likert five-point scale where 1 = strongly disagree; 5 = strongly agree***
  1. In an emergency situation, obtaining informed consent from a traumatically injured patient is important.
  2. In every decision, surgeons should seek to obtain informed consent from the patient or surrogate whenever possible.
  3. Patients cannot be expected to make informed decisions in the midst of a trauma resuscitation and therefore the wishes of trauma patients should not be respected.
  4. A central role of the trauma surgeon is to drive focus attention on the problem of violence in many environments.
  5. The trauma surgeon's expertise is in the management of traumatic injuries and trauma surgeons should not try to influence public opinion about social issues.
  6. Since traumatic injuries reflect social issues, trauma surgeons bear responsibility for focusing on changing those social issues that lead to traumatic injuries.
  7. The formal attention to ethics should be an important component of the training and education of trauma surgeons.
  8. I received adequate education in ethics during my training and education to become a trauma surgeon.
  9. I would have liked to receive more formal ethics teaching during my training to become a trauma surgeon.
  10. Ethics should be part of the trauma surgical curriculum
  11. Philosophy may help when it comes to trauma training
  12. Ethics influences decision-making
  13. Transparency is a key value that a trauma leader should employ
  14. Information management is a key value that a trauma leader should employ
  15. A trauma leader is able to identify eventual ethical breaches
  16. When ethical issues arise, it is clear how a trauma leader should behave
  17. There are situations in which a patient should be resuscitated even when there is no hope of survival so that the option of organ donation can be preserved.
  18. Sometimes a trauma surgeon should weigh the potential benefit of using resources to treat an injured patient against the likelihood of success.
  19. Trauma surgeons' responsibility to their patients should take precedence over all other societal concerns.
  20. Public health should always come first
- **20. How would you define an ethical Trauma Leader?**
  ***please type***
- **21. Which do you think are the main difficulties for Trauma Teams to gather together?**
  ***please type***

* Please note that the survey is part of the WSES “Team Dynamics I” initiative. Part of the questions are analyzed in Cobianchi L, Dal Mas F, Massaro M, Fugazzola P, Coccolini F, Kluger Y, et al. Team dynamics in emergency surgery teams: results from a first international survey. World J Emerg Surg. 2021;16:47.

The survey research protocol can be obtained by enquiring Lorenzo Cobianchi (lorenzo.cobianchi@unipv.it) or Francesca Dal Mas (francesca.dalmas@unive.it).

## Abbreviations

WSES: World Society of Emergency Surgery
CHERRIES: Checklist for Reporting Results of Internet E-Surveys
IRB: Institutional Review Board

## Footnotes

1 When the assumptions of one-way ANOVA are not met, the Kruskal–Wallis test is used. It is a nonparametric (distribution free) test. The Kruskal–Wallis test and one-way ANOVA also look for substantial differences between continuous and categorical dependent variables (with two or more groups). In an ANOVA, we assume that the dependent variable is normally distributed and that the variance in the scores between groups is roughly equal. When using the Kruskal–Wallis test, none of these assumptions are necessary. As a result, the Kruskal–Wallis test is applicable to both continuous and ordinal dependent variables.

## References

[1] Georgiou A, Lockey DJ (2010). The performance and assessment of hospital trauma teams. Scand J Trauma Resusc Emerg Med.

[2] Cobianchi L, Dal Mas F, Massaro M, Fugazzola P, Coccolini F, Kluger Y (2021). Team dynamics in emergency surgery teams: results from a first international survey. World J Emerg Surg.

[3] Massaro M, Dal Mas F, Bontis N, Gerrard B (2019). Intellectual capital and performance in temporary teams. Manag Decis.

[4] Suah A, Angelos P (2018). How should trauma patients’ informed consent or refusal be regarded in a trauma bay or other emergency settings?. AMA J Ethics.

[5] Madani A, Gips A, Razek T, Deckelbaum DL, Mulder DS, Grushka JR (2018). Defining and measuring decision-making for the management of trauma patients. J Surg Educ.

[6] Stahel PF, Cobianchi L, Dal Mas F, Paterson-Brown S, Sakakushev BE, Nguyen C (2022). The role of teamwork and non-technical skills for improving emergency surgical outcomes: an international perspective. Patient Saf Surg.

[7] Angelos P, Devon K, Ferreres AR, McLeod R, Ellison EC (2021). A Crucial moment for reflection on the importance of ethical leadership in academic medicine. Ann Surg..

[8] Cobianchi L, Dal Mas F, Angelos P (2021). One size does not fit all—translating knowledge to bridge the gaps to diversity and inclusion of surgical teams. Ann Surg.

[9] Scarlet S (2018). Caring for the wounded—the ethics of trauma surgery. AMA J Ethics.

[10] Williams-Karnesky RL, Kashyap M, Courtney C, Park C, Ritter KA, Hanke R, et al. Shoring up the pipeline: Increasing diversity in surgery by enhancing equity and inclusion in the surgical learning environment. Bulletin of the American College of Surgeons. 2021. Available from: https://bulletin.facs.org/2021/01/shoring-up-the-pipeline-increasing-diversity-in-surgery-by-enhancing-equity-and-inclusion-in-the-surgical-learning-environment/#:~:text=Arguably delayed%2C the importance of,1

[11] ASA Ensuring Equity (2018). Diversity, and inclusion in academic surgery.

[12] Gardner AK, Harris TB (2020). Beyond numbers: achieving equity, inclusion, and excellence. Ann Surg.

[13] West MA, Hwang S, Maier RV, Ahuja N, Angelos P, Bass BL (2018). Ensuring equity, diversity, and inclusion in academic surgery: an american surgical association white paper. Ann Surg.

[14] Eysenbach G (2004). Improving the quality of web surveys: the checklist for reporting results of internet E-surveys (CHERRIES). J Med Internet Res.

[15] Woltz S, Krijnen P, Pieterse AH, Schipper IB (2018). Surgeons’ perspective on shared decision making in trauma surgery. A national survey. Patient Educ Couns.

[16] Reichert M, Sartelli M, Weigand MA, Doppstadt C, Hecker M, Reinisch-Liese A (2020). Impact of the SARS-CoV-2 pandemic on emergency surgery services—a multi-national survey among WSES members. World J Emerg Surg.

[17] Rese A, Kopplin CS, Nielebock C (2020). Factors influencing members’ knowledge sharing and creative performance in coworking spaces. J Knowl Manag.

[18] Massaro M, Bardy R, Lepeley MT, Dal Mas F. Intellectual capital development in Business Schools. The role of “soft skills” in Italian Business Schools. In: Proceedings of the 5th European conference on intellectual capital. Academic Publishing Limited; 2014. p. 1–8.

[19] Dal Mas F, Garcia-Perez A, Sousa MJ, Lopes da Costa R, Cobianchi L (2020). Knowledge translation in the healthcare sector. A structured literature review. Electron J Knowl Manag.

[20] Angelos P (2020). Interventions to improve informed consent perhaps surgeons should speak less and listen more. JAMA Surg.

[21] Gwinnutt CL, Mountain A, Goode P (2021). European trauma course: the team approach.

[22] R Development Core Team. The R Manuals [Internet]. R. 2021 [cited 2021 Mar 12]. Available from: https://cran.r-project.org/manuals.html.

[23] Ferguson Bryan A, Milner R, Roggin KK, Angelos P, Matthews JB (2020). Unknown unknowns: surgical consent during the COVID-19 pandemic. Ann Surg.

[24] Yule S, Smink DS (2020). Non-Technical Skill Countermeasures for Pandemic Response. Ann Surg.

[25] Cobianchi L, Dal Mas F, Peloso A, Pugliese L, Massaro M, Bagnoli C (2020). Planning the full recovery phase: an antifragile perspective on surgery after COVID-19. Ann Surg.

[26] Dal Mas F, Bagarotto EM, Cobianchi L, Lepeley MT, Beutell N, Abarca N, Majluf N (2021). Soft skills effects on knowledge translation in healthcare. Evidence from the field. Soft skills for human centered management and global sustainability.

[27] Briggs A, Raja AS, Joyce MF, Yule SJ, Jiang W, Lipsitz SR (2015). The role of nontechnical skills in simulated trauma resuscitation. J Surg Educ.

[28] Sade RM, Kavarana MN (2017). Surgical ethics: today and tomorrow. Future Cardiol.

[29] Logghe HJ, Rouse T, Beekley A, Aggarwal R (2018). The evolving surgeon image. AMA J Ethics.

[30] Bethlehem J (2010). Selection bias in web surveys. Int Stat Rev.

[31] Nembhard IM, Singer SJ, Shortell SM, Rittenhouse D, Casalino LP (2012). The cultural complexity of medical groups. Health Care Manag Rev.

